# Unsuccessful and Successful Clinical Trials in Acute Respiratory Distress Syndrome: Addressing Physiology-Based Gaps

**DOI:** 10.3389/fphys.2021.774025

**Published:** 2021-11-30

**Authors:** Jesús Villar, Carlos Ferrando, Gerardo Tusman, Lorenzo Berra, Pedro Rodríguez-Suárez, Fernando Suárez-Sipmann

**Affiliations:** ^1^CIBER de Enfermedades Respiratorias, Instituto de Salud Carlos III, Madrid, Spain; ^2^Multidisciplinary Organ Dysfunction Evaluation Research Network (MODERN), Research Unit, Hospital Universitario Dr. Negrín, Las Palmas de Gran Canaria, Spain; ^3^Keenan Research Center at the Li Ka Shing Knowledge Institute, St. Michael’s Hospital, Toronto, ON, Canada; ^4^Department of Anesthesiology and Critical Care, Hospital Clinic, Barcelona, Spain; ^5^Institut d’Investigacions Biomèdiques August Pi i Sunyer (IDIBAPS), Hospital Clinic, Barcelona, Spain; ^6^Department of Anesthesiology, Hospital Privado de Comunidad, Mar del Plata, Argentina; ^7^Harvard Medical School, Boston, MA, United States; ^8^Department of Anesthesia, Critical Care and Pain Medicine, Massachusetts General Hospital, Boston, MA, United States; ^9^Department of Thoracic Surgery, Hospital Universitario Dr. Negrín, Las Palmas de Gran Canaria, Spain; ^10^Intensive Care Unit, Hospital Universitario La Princesa, Madrid, Spain; ^11^Hedenstierna Laboratory, Department of Surgical Sciences, Anesthesiology and Critical Care, Uppsala University Hospital, Uppsala, Sweden

**Keywords:** acute respiratory distress syndrome, clinical trials, neuromuscular blockade, prone ventilation, high-frequency ventilation, positive end-expiratory pressure, extracorporeal oxygenation, anti-inflammatory drugs

## Abstract

The acute respiratory distress syndrome (ARDS) is a severe form of acute hypoxemic respiratory failure caused by an insult to the alveolar-capillary membrane, resulting in a marked reduction of aerated alveoli, increased vascular permeability and subsequent interstitial and alveolar pulmonary edema, reduced lung compliance, increase of physiological dead space, and hypoxemia. Most ARDS patients improve their systemic oxygenation, as assessed by the ratio between arterial partial pressure of oxygen and inspired oxygen fraction, with conventional intensive care and the application of moderate-to-high levels of positive end-expiratory pressure. However, in some patients hypoxemia persisted because the lungs are markedly injured, remaining unresponsive to increasing the inspiratory fraction of oxygen and positive end-expiratory pressure. For decades, mechanical ventilation was the only standard support technique to provide acceptable oxygenation and carbon dioxide removal. Mechanical ventilation provides time for the specific therapy to reverse the disease-causing lung injury and for the recovery of the respiratory function. The adverse effects of mechanical ventilation are direct consequences of the changes in pulmonary airway pressures and intrathoracic volume changes induced by the repetitive mechanical cycles in a diseased lung. In this article, we review 14 major successful and unsuccessful randomized controlled trials conducted in patients with ARDS on a series of techniques to improve oxygenation and ventilation published since 2010. Those trials tested the effects of adjunctive therapies (neuromuscular blocking agents, prone positioning), methods for selecting the optimum positive end-expiratory pressure (after recruitment maneuvers, or guided by esophageal pressure), high-frequency oscillatory ventilation, extracorporeal oxygenation, and pharmacologic immune modulators of the pulmonary and systemic inflammatory responses in patients affected by ARDS. We will briefly comment physiology-based gaps of negative trials and highlight the possible needs to address in future clinical trials in ARDS.

## Background

The acute respiratory distress syndrome (ARDS) is a severe form of acute hypoxemic respiratory failure. Caused by an intense direct (pulmonary) or indirect (systemic) inflammatory insult to the alveolar-capillary membrane, it is characterized by the presence of diffuse, non-cardiogenic, high-permeability, protein-rich pulmonary edema, and hypoxemia unresponsive to the application of high inspiratory concentrations of oxygen (FiO_2_) (Villar, 2011). The use of mechanical ventilation (MV) is the standard supportive therapy of patients with ARDS. Since the publication in 2000 of the milestone paper by the ARDS Network (Acute Respiratory Distress Syndrome Network, Brower et al., 2000), the aim of MV is to achieve adequate gas-exchange avoiding damaging the lungs by using physiological tidal volumes (VT) of 4–8 ml/kg predicted body weight (PBW), preventing alveolar collapse with positive end-expiratory pressure (PEEP), limiting end-inspiratory plateau pressure (Pplat) to less than 30 cmH_2_O, and limiting FiO_2_ to maintain an adequate PaO_2_. These essential elements are the main components of the framework for lung-protective MV.

## Introduction

Most ARDS patients improve their oxygenation, as assessed by the ratio between the arterial partial pressure of oxygen (PaO_2_) and FiO_2_ (PaO_2_/FiO_2_), disease-specific treatment and the application of adequate levels of PEEP. There is no typical ARDS patient. Over the years, hypoxemia has become an infrequent cause of death in ARDS (Slutsky et al., 2016). Physiologically, we are unaware of data linking a specific PaO_2_/FiO_2_ to predictable morphological changes in the alveolar-capillary membrane at ARDS onset. Recent evidence has shown an association between severity of lung damage and prediction of outcome when the PaO_2_/FiO_2_ is evaluated at 24 h under standardized ventilator settings using an enrichment strategy (Villar et al., 2019). We lack a standard definition for refractory or persisting hypoxemia, as a predetermined PaO_2_ value under a particular FiO_2_ and PEEP for a specific time-period. In this review, we will consider hypoxemia for enrolling patients into clinical trials when the PaO_2_/FiO_2_ is ≤ 200 mmHg on MV and PEEP ≥ 5 cmH_2_O, in agreement with the Berlin criteria for moderate-to-severe ARDS (Ranieri et al., 2012). The purpose of this brief review is to summarize the current knowledge based on a number of major randomized controlled trials (RCTs) performed in ARDS patients on MV evaluating interventions applied during the acute phase and published since 2010, independent of whether they reported benefits or not in the primary or secondary outcomes when testing the experimental intervention. Those RCTs were conducted with the aim to improve oxygenation and mortality while the patient was in the intensive care unit (ICU) or after being discharged from ICU. We will discuss the physiology-based gaps of negative trials and highlight the possible needs to address in future trials design in ARDS.

## Review of Randomized Controlled Trials

Since 2010, 14 major RCTs (Papazian et al., 2010; Gao Smith et al., 2012; Ferguson et al., 2013; Guerin et al., 2013; Young et al., 2013; McAuley et al., 2014; Kacmarek et al., 2016; Working group for the Alveolar Recruitment for Acute Respiratory Distress Syndrome Trial (Art) Investigators et al., 2017; Combes et al., 2018; Beitler et al., 2019; Hodgson et al., 2019; The National Heart, Lung, and Blood Institute Petal Clinical Trials Network, Moss et al., 2019; Ranieri et al., 2020; Villar et al., 2020) have been published in patients with ARDS (Table 1). In those trials, patients received invasive MV and tested different adjunctive or rescue therapies for improving oxygenation, or different MV approaches to titrate PEEP, or several anti-inflammatory drugs for attenuating the pulmonary and systemic inflammatory responses.

**TABLE 1:** Successful and unsuccessful randomized clinical trials since 2010 in ventilated patients with acute respiratory distress syndrome (ARDS).

References publication year	Trial name	Study period, No. ICUs, Country	Criteria for enrollment	Patients	Intervention	Major findings	Remarks
Papazian et al. (2010)	ACURASYS	2006–2008 (24 months) 20 ICUs, France	MV, PaO_2_/FiO_2_ < 150 with PEEP ≥ 5 for < 48 h	340	Neuromuscular blockers (cisatracurium)	Improved adjusted 90-day mortality and VFDs	Control group was deeply sedated
Gao Smith et al. (2012)	BALTI-2	2006–2010 (40 months) 46 ICUs, UK	MV, within 72 h of ARDS onset (AECC criteria)	326	Salbutamol	Salbutamol worsen outcomes	Concerns for use of non-protective MV
Guerin et al. (2013)	PROSEVA	2008–2011 (41 months) 27 ICUs France, Spain	MV < 36 h, PaO_2_/FiO_2_ < 150 on FiO_2_ ≥ 0.6 confirmed at 12–24 MV	466	Prone positioning for at least 12 h/daily	Decreased 28-day and 90-day mortality	Currently, it is standard of care in severe ARDS
Ferguson et al. (2013)	OSCILLATE	2009–2012 (38 months) 39 ICUs in Canada, United States, Saudi Arabia, Chile, India	MV, PaO_2_/FiO_2_ ≤ 200 on FiO_2_ ≥ 0.5 and PEEP ≥ 10	548	High-frequency oscillation ventilation (HFOV)	Increased ICU and hospital mortality	Increasing harm from HFOV at higher PaO_2_/FiO_2_
Young et al. (2013)	OSCAR	2007–2012 (55 months) 29 ICUs, UK	MV, AECC criteria, PaO_2_/FiO_2_ ≤ 200 on PEEP ≥ 5	795	High-frequency oscillation (HFOV)	No change in 30-day mortality	HFOV increased harm at higher PaO_2_/FiO_2_
McAuley et al. (2014)	HARP-2	2010–2014 (39 months) 40 hospitals in UK and Ireland	MV, < 48 h from ARDS onset, PaO_2_/FiO_2_ ≤ 300 (AECC criteria)	540	Simvastatin	No effects on outcomes	Concerns for use of non-protective MV
Kacmarek et al. (2016)	OLA	2007–2013 (59 months) 20 ICUs in Spain, South Korea, Brazil	MV, PaO_2_/FiO_2_ ≤ 200 (AECC criteria). At 24 h, PaO_2_/FiO_2_ ≤ 200 on FiO_2_ ≥ 0.5 and PEEP ≥ 10	200	Open lung approach (lung recruitment and PEEP titration)	Increased oxygenation and decreased driving pressure. No change in ICU mortality	Prognostic enrichment for enrollment at 12–36 h after ARDS onset
Working group for the Alveolar Recruitment for Acute Respiratory Distress Syndrome Trial (Art) Investigators et al. (2017)	ART	2011–2017 (65 months) 120 ICUs in Brazil, Argentina, Colombia, Italy, Poland, Portugal, Malaysia, Spain, Uruguay	MV, ARDS (AECC criteria) < 72 h, enrollment if PaO_2_/FiO_2_ ≤ 200 on PEEP ≥ 10 and FiO_2_ = 1 for 30 min	1,010	Open lung approach (lung recruitment and PEEP titration)	Increased 28-day and 6-month mortality. Decreased VFDs. Increased risk of barotrauma	Concerns with study design, methodology, data analysis, and differences in health care systems
Combes et al. (2018)	EOLIA	2012–2017 (55 months) 23 ICUs in France, Canada, United States	Very severe ARDS: PaO_2_/FiO_2_ < 50 for > 3 h; or PaO_2_/FiO_2_ ≤ 80 for > 6 h; or pH < 7.25 with PaCO_2_ ≥ 60 for > 6 h	249	Extracorporeal membrane oxygenation (ECMO)	No significant benefit in 60-day mortality	Control group included crossover to ECMO in 28% patients
The National Heart, Lung, and Blood Institute Petal Clinical Trials Network, Moss et al. (2019)	ROSE	2016–2018 (28 months) 48 hospitals in United States	MV, PaO_2_/FiO_2_ < 150 with PEEP ≥ 8 for < 48 h	1,006	Neuromuscular blockers (cisatracurium)	No significant benefit in 90-day mortality	Control group with lighter sedation
Beitler et al. (2019)	EPVent-2	2012–2017 (59 months) 14 hospitals in United States	MV, PaO_2_/FiO_2_ ≤ 200 within 36 h ARDS onset (Berlin criteria)	200	Esophageal pressure-guided for titrating PEEP	No significant benefit in 28-day mortality and VFDs	Median PEEP levels was similar in both groups over time
Hodgson et al. (2019)	PHARLAP	2012–2017 (59 months) 35 ICUs in Australia, New Zealand, Ireland, Saudi Arabia, UK	MV < 72 h, ARDS or other types of respiratory failure with PaO_2_/FiO_2_ ≤ 200 on PEEP ≥ 5	113	Lung recruitment maneuvers with PEEP titration	No benefits in VFDs or ICU/hospital mortality	Small sample size, PEEP titration used SpO_2_, and treatment crossovers
Ranieri et al. (2020)	INTEREST	2015–2017 (25 months) 74 ICUs, 8 European countries	MV, PaO_2_/FiO_2_ ≤ 200 PEEP ≥ 5 (Berlin criteria) within 24 h	301	Interferon β-1a	No significant benefit in 28-day mortality and VFDs	Higher-than-expected use of corticosteroids
Villar et al. (2020)	DEXA-ARDS	2013–2018 (69 months) 17 ICUs, Spain	MV, PaO_2_/FiO_2_ ≤ 200 at ARDS onset; at 24 h, PaO_2_/FiO_2_ ≤ 200 on FiO_2_ ≥ 0.5 and PEEP ≥ 10	277	Dexamethasone	Increased VFDs. Decreased 60-day mortality	Prognostic enrichment for enrollment at 24 h of ARDS onset

*AECC, American-European Consensus Conference; ICU, intensive care unit; MV, mechanical ventilation; PEEP, positive end-expiratory pressure; SpO_2_, oxygen saturation; UK, United Kingdom; VFDs, ventilator-free days.*

### Muscle Paralysis

There is sufficient evidence showing that MV can initiate or aggravate lung injury, a concept labeled as ventilator-induced lung injury (VILI) (Slutsky and Ranieri, 2013). Many of the consequences of VILI bear a resemblance to those of ARDS. Despite limiting VT and pressures during lung-protective MV, ARDS patients could develop tidal hyperinflation during spontaneous respiratory efforts while mechanically ventilated, especially in the early phases of ARDS. VT set in the ventilator does not always correspond to the exact VT delivered to the patient, due to the contribution of inspiratory muscle efforts to inflation pressure and more importantly in the presence of double triggering, reverse triggering, and other types of patient-ventilator asynchronies, which could develop despite receiving deep analgesia or sedation. Papazian et al. (2010) conducted an RCT, the *ARDS et Curarisation Systematique* (ACURASYS) trial, to examine the hypothesis that removing spontaneous respiratory efforts would improve lung mechanics and oxygenation in patients with persistent hypoxemia. It was known that neuromuscular blockade (NMB) decreases the work of breathing and asynchronies in ARDS (Light et al., 1975). The ACURASYS trial enrolled 340 ARDS patients with moderate-to-severe ARDS (defined by the investigators as a PaO_2_/FiO_2_ < 150 mmHg on PEEP ≥ 5 cmH_2_O during the first 2 days of ARDS diagnosis). Patients were randomized to receive either cisatracurium or placebo for 48 h. Patients in the control arm were deeply sedated. The authors found that in patients assigned to NMB, the adjusted 90-day mortality was lower and ventilator free-days (VFDs) were higher than in those not receiving NMB.

The findings of the ACURASYS remained controversial for almost a decade for two reasons: the Kaplan-Meir survival curves of the two treatment arms separated only after 14 days of randomization, and, most importantly, the 90-day all-cause mortality only reached statistical significance with intensity adjustment (Yegneswaran and Murugan, 2011). Thus, additional validation of this trial was required. A RCT termed “*Reevaluation Of Systemic Early neuromuscular blockade (ROSE) trial*” (The National Heart, Lung, and Blood Institute Petal Clinical Trials Network, Moss et al., 2019) was unable to validate ACURASYS results. The ROSE trial was powered for evaluating the efficacy and safety of NMB in decreasing 90-day mortality. The trial stopped for futility when 1006 ARDS patients were enrolled. Although this trial was not an exact replication of ACURACYS (since both arms of the trial received PEEP ≥ 8 cmH_2_O, and the control arm received lighter sedation targets), there were no differences between groups in the rate of barotrauma and in the number of VFDs, and 90-day mortality was virtually identical in the two groups.

What are the implications of these two RCTs? First, according to these results routine use of NMB agents are not recommended in moderate-to-severe ARDS patients. Contrary to expectations, the prevalence of patient-ventilator asynchronies in the form of reverse triggering (it occurs when a breath delivered by the ventilator triggers diaphragm contraction, initiating an assisted breath –the reverse of what occurs during assisted MV) increases with deeper sedation levels (Bourenne et al., 2019). Second, from a pathophysiological point of view, there is a basis to use NMB in any patient with ARDS when after ensuring an adequate ventilation and sedation, the patient has a ventilatory pattern that could promote VILI. Therefore, current data suggest that NMB agents can be used when they are physiologically and clinically indicated (Slutsky and Villar, 2019).

### Prone Ventilation

Patho-physiologically and histo-pathologically, ARDS is a heterogeneous inflammatory process produced by a variety of insults with collapsed and consolidated areas mainly in the dependent regions, and more healthy units in the non-dependent regions (Villar, 2011). Thus, recruitability of alveolar spaces in ARDS lungs with PEEP is also heterogeneous, both between patients and within the lungs. Changes in body posture could have marked effects on pulmonary function in patients with acute respiratory failure. As normal practice, the critically ill patient is cared for in supine. The usual reduction in function residual capacity (FRC) when adopting the supine position is increased in ARDS ventilated patients, resulting in ventilation-perfusion mismatching and a fall in the PaO_2_. Five mechanisms have been proposed to explain the improved oxygenation during prone positioning: (i) increased FRC, (ii) changes in regional diaphragm motion, (iii) improved ventilation-perfusion matching due to a redistribution of regional ventilation to dorsal regions of the lung, (iv) improved clearance of secretions, and (v) removal of the weight of the heart from the lung (Lamm et al., 1994).

Prone ventilation is considered an adjunctive intervention to the ventilatory management of ARDS patients with refractory hypoxemia. Prone ventilation can be performed safely if ICU teams are adequately trained. After several studies reported conflicting results about the efficacy of prone ventilation in persistent hypoxemia, a large RCT, the “*Proning Severe ARDS patients” (PROSEVA)* trial (Guerin et al., 2013), reported survival benefit in moderate-to-severe ARDS. In that trial, 466 patients with persistent ARDS (as defined by a PaO_2_/FiO_2_ < 150 mmHg with FiO_2_ ≥ 0.6 and PEEP ≥ 5 cmH_2_O) were randomly assigned to prone position for at least 16 h or to supine position. Patients in both groups were mechanically ventilated following the low PEEP-FiO_2_ table from the ARDSnet trial (Acute Respiratory Distress Syndrome Network, Brower et al., 2000). All-cause mortality at 28 days was 33% in the supine group and 16% in the prone group, a highly significant difference that persisted 90 days after randomization.

Proponents of prone ventilation advise that the approach of the PROSEVA trial was a modification of a technique that finally became right at a time when patients were mechanically ventilated with low VT (Beitler et al., 2014). However, although prone positioning improves oxygenation in ARDS patients and could help in recruiting lung regions, there is controversy over its use in clinical practice (Villar et al., 2014). The large treatment effect seems too good to be true: a 28-day mortality of 16% is the lowest, ever reported in a trial, or in any observational study on ARDS. Furthermore, patients assigned to the supine group were mechanically ventilated during the first 72 h with low levels of PEEP (9 ± 3 cmH_2_O). Therefore, as with NMB, an additional RCT is required to confirm these findings. Such a trial should guarantee that the control group will receive a higher PEEP approach.

### High Frequency Oscillatory Ventilation

Knowledge on the mechanisms and importance of VILI has advanced over the years. Theoretically, from the perspective of lung-protective ventilation, high frequency oscillatory ventilation (HFOV) would be an ideal mode of ventilation for ARDS patients (Ferguson et al., 2007). By definition, it accomplishes adequate gas-exchange by providing very small VT that are typically 1–3 ml/kg, often less than the anatomic dead space, at frequencies from 3 to 15 cycles per second at a constant mean airway pressure. Since HFOV incorporates fewer and simpler controls that are not interrelated, it is an easier technique than conventional MV. However, there is a lack of evidence showing that HFOV is less harmful that using MV with low VT, moderate-to-high PEEP, and limitation of Pplat.

A critical examination of two RCTs comparing HFOV with lung-protective MV published in 2013 (enrolling 1343 ARDS patients) demonstrated no benefits of HFOV. In the OSCILLATE (*OSCILLation for Acute respiratory distress syndrome Treated Early*) trial (Ferguson et al., 2013), the investigators stopped the trial after 548 of the 1,200 planned patients were randomized. Patients were eligible for inclusion if they had a PaO_2_/FiO_2_ ≤ 200 mmHg on FiO_2_ ≥ 0.5. However, only patients with a PaO_2_/FiO_2_ ≤ 200 after 30 min of assessment on a VT of 6 ml/kg PBW, PEEP ≥ 10 and FiO_2_ of 0.6 were randomized. Patients in the HFOV group received HFOV after lung recruitment with an “open lung approach” (OLA) consisting in the application of a recruitment maneuver (RM) of 40 cmH_2_O of pressure for 40 s for a median of 3 days. However, almost 15% of patients in the control group received HFOV as a rescue therapy. The absolute hospital mortality was 12% higher in the HFOV group (relative risk of death 1.33, *p* = 0.005). The relatively high mortality in the HFOV group could be explained by the higher prevalence of hemodynamic compromise (hypotension) after the initiation of HFOV, contributing to extrapulmonary organ dysfunction, and by increased use of sedative agents. In the OSCAR (*OSCillation in ARDS*) trial (Young et al., 2013), the study design was more pragmatic. The authors randomized 795 patients with a PaO_2_/FiO_2_ ≤ 200 on PEEP ≥ 5 cmH_2_O. Patients in the HFOV arm were ventilated by increasing mean airway pressure and FiO_2_. The control group was treated according to local practice in participating ICUs, applying the low PEEP-FiO_2_ table from the ARDSnet trial (Acute Respiratory Distress Syndrome Network, Brower et al., 2000). There were no differences in 30-day mortality between the groups.

What are the implications of these two trials? In a recent meta-analysis of 1,552 ARDS patients from four RCTs (Meade et al., 2017), including those from OSCILLATE and OSCAR trials, the investigators reported a significant interaction between PaO_2_/FiO_2_ at randomization and the effects of HFOV, revealing increased harm from HFOV at higher PaO_2_/FiO_2_. Although HFOV still have a place as a rescue therapy, especially in centers without access or experience in extracorporeal oxygenation, this meta-analysis strongly question its future use in ARDS (Vincent, 2017).

### Recruitment Maneuvers and Transpulmonary Pressure

Therapeutic variation in the distribution of inspired gas for attenuating VILI is the foundation of both prone ventilation and RMs. With the use of computed tomography (CT), it was discovered that radiographically some lung regions in ARDS look relatively normal but other areas are partially collapsed and do not participate in gas-exchange. RMs are applied to reopen collapsed alveolar units and to attenuate the injurious effects of repetitive opening and closing of alveoli. In general, ARDS is a heterogeneous injury in three lung compartments (Nieman et al., 2020): (i) normal alveoli that are inflated at end of expiration –and is referred to as the “baby lung” (Gattinoni and Pesenti, 2005); (ii) alveoli that are collapsed and/or fluid filled; (iii) and alveoli that are in between normal and unstable, and that open and collapse with every breath. Of note, the “baby lung” concept led to the understanding of potential interaction between MV settings and outcome, and to the frequent use of CT as a standard tool for a more precise ventilatory management in ARDS patients. As a result, lung-protective MV is constrained by ventilating this heterogeneous lung to avoid VILI, by protecting the baby lung without overdistending the compliant lung, and by stabilizing the lung using PEEP. Compliance is a property that describes lung distensibility and is calculated as the change in lung volume divided by the change in pressure. Lung compliance of the respiratory system is calculated as the VT divided by the transpulmonary pressure, the pressure across the lung (or alveolar pressure minus pleural pressure) (Kacmarek et al., 2021). Atelectatic areas of the lungs can be expanded by a brief application of high transpulmonary pressure, followed by a PEEP level that maintains open the new re-aerated region (Suárez-Sipmann et al., 2007). PEEP prevents lung collapse at end expiration. Although there are three commonly used RMs (sighs, sustained inflations, and extended sighs) (Guerin et al., 2011), none of these three maneuvers are currently recommended. In fact, none of the above trials used those maneuvers, but favored the use of incremental PEEP steps maintaining a constant driving pressure (Pplat minus PEEP) in a pressure-controlled mode until the recruitment pressures are reached.

There is controversy about the outcome benefits of RMs in patients with ARDS. In a pilot RCT in 200 patients with persistent ARDS (Kacmarek et al., 2016) comparing the ARDSnet protocol (Acute Respiratory Distress Syndrome Network, Brower et al., 2000) with an OLA approach involving RMs and a decremental PEEP trial for identifying the level of PEEP associated with maximum dynamic compliance, OLA improved oxygenation and lung mechanics without harmful effects on all-cause mortality at 60 days, VFDs, or barotrauma. In a large RCT (Working group for the Alveolar Recruitment for Acute Respiratory Distress Syndrome Trial (Art) Investigators et al., 2017), the *Alveolar Recruitment for acute respiratory distress syndrome Trial (ART)* conducted at 120 ICUs in 9 countries (mostly from Brazil) and involving 1010 patients with moderate to severe ARDS, RMs and PEEP titration according to best respiratory system compliance did not show reduced 28-day mortality when compared to patients treated with lower levels of PEEP. By contrary, patients in the RMs group had higher 6-month mortality, lower VFDs, and increased risk of barotrauma. After the publication of the negative results of the ART trial, the *Permissive Hypercapnia, Alveolar Recruitment, and Low Airway Pressure (PHARLAP)* trial (Hodgson et al., 2019) evaluating the effects of an OLA strategy with RMs vs. lung-protective MV was stopped very early when only 113 patients with moderate-to-severe ARDS were randomized (of a planned 340 patients). This small sample size trial (57 patients in the RM group vs. 56 in the control group) had no power to identify differences in VFDs, risk of barotrauma, or 180-day mortality.

Although the findings of the ART trial do not support the routine application of RMs, major concerns about study design, methodology, data analyses, and important differences with health care systems in participating countries, provided solid reasons to distrust the results of the trial and their generalizability to other settings (Villar et al., 2017). Patho-physiologically, there is a rationale for using RM and PEEP titration in ARDS since the ART results are in conflict with previous physiological and clinical data. There is a need for another RCT designed and implemented more appropriately to examine that the principle that “never give the lungs a chance to collapse” (Villar et al., 2017) is associated with better clinical benefits, although all-cause fatality rate might not be reduced further.

A newer technique for titrating PEEP is optimizing the transpulmonary pressure at end of expiration (PEEP minus pleural pressure) (Talmor et al., 2008). Pleural pressure is estimated *via* esophageal manometry employing the measurement of esophageal pressure at the end of expiration, as a surrogate estimate of pleural pressure. It differs among patients with hypoxemic acute respiratory failure, suggesting that lung and chest wall mechanics contribute to respiratory system mechanics, as measured by the mechanical ventilator (Kacmarek et al., 2021). In general, the transpulmonary end-expiratory pressure is equal to zero: the more negative the transpulmonary pressure, the greater the collapse caused by a reduction in lung compliance. A negative transpulmonary pressure reflects that the forces trying to collapse the lung are stronger than the forces maintaining the lung open. This process may reverse if the end-expiratory transpulmonary pressure becomes zero or positive (Fumagalli et al., 2017). That is the main rationale for applying PEEP. PEEP increases the alveolar pressure. When alveolar pressure is equal to or exceeds pleural pressure, the resulting transpulmonary pressure is positive. This mechanism reduces lung collapse if the lung is opened before applying PEEP. That is why performing a RM and setting the appropriate level of PEEP by a decremental titration, augments FRC, decreases atelectasis, improves oxygenation, and increases compliance when compared with the same incremental PEEP level without recruitment (Pirrone et al., 2016).

A multicenter trial (EPVent-2) (Beitler et al., 2019) to validate preliminary results of a previous pilot study on esophageal pressure-guided ventilation (Talmor et al., 2008) was conducted in 200 patients with moderate-to-severe ARDS and examined whether PEEP titration guided by esophageal pressure is more effective than PEEP using a modified PEEP-FiO_2_ table adopted from the OSCILLATE trial (Ferguson et al., 2013) using higher PEEP values. The primary outcome was a composite score including mortality and VFDs, calculated in a way that death was a worse outcome than fewer days free of MV. However, values for esophageal end-expiratory pressure, PEEP, Pplat, driving pressure, and PaO_2_/FiO_2_ were similar between both groups within the first 7 days. Although patients allocated to esophageal pressure-guided PEEP received fewer rescue therapies, the primary endpoint was not different between groups. Although for the investigators these findings did not support the use of PEEP titration using esophageal manometry, there are several reasons that could explain a lack of benefits of this trial (Suarez-Sipmann et al., 2019). First, in the multicenter EPVent-2 trial, participating centers had a wide range of expertise in implementing this novel method, as contrary to the high expertise of investigators in the previous EPVent trial. Second, the study design was not a true validation of the tested hypothesis in the EPVent since the EPVent-2 incorporated the PEEP-FiO_2_ table using much higher PEEP levels, and oxygenation was not the primary outcome. This led to similar mean values of PEEP and transpulmonary pressures in both groups, suggesting that, on average, both groups were ventilated similarly! Third, the trial was underpowered: the calculation of the trial sample size was based on an overestimated 22% absolute difference in 28-day mortality. Fourth, a major critique relates to the PEEP titration method. Linking the transpulmonary pressure to the oxygenation level by a non-physiological PEEP-FiO_2_ table was an erroneous decision to assess the effects of PEEP in the esophageal manometry group. Changes in oxygenation could not parallel changes in lung mechanics. For example, in the control group patients requiring FiO_2_ ≥ 0.5 could be ventilated with PEEP > 16 cmH_2_O, a level that exceeds by far what is considered usual care. On the other hand, there is no guarantee that esophageal pressure recordings were checked for quality control in most participating centers. Fifth, it is plausible that the trial was biased in favor of the control group since both groups were monitored with an esophageal catheter.

A recent *post-hoc* reanalysis of the EPVent-2 trial by the same authors (Sarge et al., 2021) showed that the effect of PEEP strategy on mortality depended on pre-intervention severity of multiorgan dysfunction. Esophageal manometry-guided PEEP was associated with lower mortality among patients with less severe multiorgan dysfunction. Intriguingly, the higher end-inspiratory transpulmonary pressure, a marker of tidal overdistension, was independently correlated with risk of circulatory shock. Future clinical trials should be designed to consider baseline both, esophageal pressure at the end of expiration (to minimize the development of atelectasis) and esophageal pressure at the end of inspiration (to minimize the development of overdistension).

### Extracorporeal Membrane Oxygenation

Oxygenation using extracorporeal life support was initially used in patients with severe acute respiratory failure in whom it was impossible to provide adequate gas-exchange by MV (Egan et al., 1988). MV is dependent on the presence of functional lung units for gas diffusion. However, when the number of functional alveoli is markedly reduced, MV is unable to sustain gas-exchange. In those cases, replacing the alveolar gas-exchange by extracorporeal membrane oxygenation (ECMO) can substantially reduce VT, respiratory rate, and FiO_2_, and the risk of developing VILI. Most adult ECMO for respiratory support are performed with a veno-venous technique (Munshi et al., 2019). To deliver gas-exchange during ECMO, part of the cardiac output goes through the extracorporeal circuit *via* the femoral, saphenous, or jugular veins. CO_2_ is removed by the ECMO circuit while MV is applied at low respiratory rates, high levels of PEEP, and Pplat below 30 cmH_2_O by applying very low VTs.

Today, ECMO equipment are simpler, cheaper, and safer. Despite advances in extracorporeal life support and worldwide clinical use of ECMO, the results of a recent RCT in ARDS patients (Combes et al., 2018) has led to a diminished enthusiasm for using it in severe ARDS. Referred to as the EOLIA (*ECMO to rescue Lung Injury in severe ARDS*) trial, this international RCT examined the effects of early use of ECMO in patients with very severe ARDS (Table 1). Patients assigned to the control group received MV, and the use of NMB agents, prone positioning, RMs, inhaled nitric oxide, or prostacyclin were strongly encouraged for oxygenation objectives by protocol. Patients assigned to ECMO underwent percutaneous veno-venous cannulation. The data safety monitoring board of the study decided to stop the trial after 75% of the planned patient population had been enrolled because the lower boundary of the predefined stopping rule for futility (defined as < 20% absolute risk reduction in mortality at 60 days) was achieved. The planned sample size was 331 patients, but the trial was terminated when 249 patients were randomized. In retrospect, a 20% absolute risk reduction is an unreasonable very large effect size when calculating sample size. Had the trial been designed with less strict stopping rules and continued to full enrollment, EOLIA may would have reached statistical significance. A major flaw of the trial was that 28% of patients allocated to the control arm (lung protective MV) crossed over to receive ECMO after randomization. This high crossover rate makes it extremely problematic to draw definitive conclusions about the benefits of ECMO in ARDS patients with very severe hypoxemia. Furthermore, the crossover of patients randomized to ECMO in participating ECMO centers could be seen as a weakness since ECMO is not available in most cities of modern countries for treating very severe hypoxemic ARDS.

Despite the results of the EOLIA trial, many clinicians still believe that there is a role for ECMO in severe ARDS patients with single organ failure and potentially reversible pulmonary dysfunction when MV and other adjunctive therapies have failed. Ideally, a comparison among patients who crossed over to ECMO with patients with similar severity randomized to the ECMO group would be an appropriate task to do. However, no such subsets of patients were identified in the ECMO group after a careful systematic review of the EOLIA trial (Munshi et al., 2019).

### Pharmacologic Modulators of the Pulmonary and Systemic Inflammatory Responses

Anti-inflammatory drugs have been tested as prophylaxis and/or treatment of ARDS. In 2019, a systematic review provided no conclusive evidence that any pharmacologic intervention could reduce mortality in ARDS patients (Santacruz et al., 2019).

In the last decade in the United Kingdom (UK), two large RCTs (7,11) evaluated pharmacologic therapies to improve outcome in ARDS patients. The BALTI-2 (*beta-2 agonist lung injury trial*) (7) investigated the effects of increasing alveolar water clearance using intravenous salbutamol. This was a multicenter, placebo-controlled, randomized trial at 46 ICUs between 2006 and 2010, where patients within 72 h of ARDS onset, as defined by the American-European Consensus Conference (AECC) criteria (Bernard et al., 1994), were allocated to receive either salbutamol or placebo for 7 days. The primary outcome was death at 28 days of randomization. The trial reported harm in the treatment group and was stopped after the second interim analysis. A total of 324 patients (161 in the salbutamol group vs. 163 in the placebo group) were analyzed. Treatment with salbutamol significantly increased 28-day mortality, and was poorly tolerated. The HARP-2 (*Hydroxymethylglutaryl-CoA reductase inhibition with simvastatin in Acute lung injury to Reduce Pulmonary dysfunction-2*) (11) study tested the use of the anti-inflammatory effects of simvastatin in ARDS. This was a multicenter, double-blind, randomized trial at 40 ICUs from UK and Ireland between 2010 and 2014, where 540 patients within 48 h of ARDS onset, as defined by AECC criteria, were randomized. The primary outcome was the number of VFDs, and secondary outcomes included mortality at 28 days. Treatment with simvastatin was safe, but it did not improve clinical outcomes.

Although the pharmacological mechanisms underlying the increased mortality in patients receiving salbutamol and the no effects of simvastatin are unclear, there are serious concerns about the approach for ventilating ARDS patients in both trials. Despite that leading investigators of both trials recommended the use of lung-protective MV, an audit (Poole et al., 2017) examining the ventilation practice in BALTI-2 and HARP-2 trials found that compliance with the ARDSnet guidelines for VT was very poor across all time points. There was no feedback to participating centers for the MV management in these studies. Baseline data was only available in 49% of patients in the BALTI-2 trial and PEEP levels were not recorded in both trials. This audit analysis revealed that considerably less than half of patients in BALTI-2 and HARP-2 trials received lung protective MV. Given the importance of low VT ventilation in improving outcomes ARDS, the results of those two pharmacologic trials need to be re-examined.

The key pathophysiological event underlying ARDS is injury to both the lung capillary endothelium and the alveolar epithelium with increased pulmonary vascular leakage. It turns out that adenosine has anti-inflammatory properties and reduces endothelial permeability. The enzyme termed cluster of differentiation 73 (CD73) is expressed on endothelial and epithelial cells, and regulates adenosine production by converting extracellular adenosine to active adenosine (Thompson et al., 2004). Since interferon (IFN) β-1a upregulates CD73, preventing vascular leakage, a pilot nonrandomized study reported that treatment with recombinant human IFN-β was associated with a reduction of 28-day mortality in patients with ARDS (Bellingan et al., 2014). In the INTEREST trial (*Efficacy and Safety of Interferon beta-1a in patients with ARDS*), a multicenter, randomized, double-blind, parallel-group in 8 countries, 301 patients with moderate or severe ARDS were randomized to treatment with 10 μg of IFN-1a once daily for 6 days or to placebo (Ranieri et al., 2020). The primary outcome was a composite score combining number of VFDs and number of deaths at 28 days. A total of 296 patients completed the trial, which did not lead to fewer deaths and VFDs when compared to the placebo group. Apart from the lack of efficacy, the trial has two important limitations: (i) the study was underpowered since no enrichment approach was considered to enroll patients; (ii) more than one third of patients received steroids, which inhibit the effects of IFN-β-1a signaling, raising the possibility that the treatment with IFN- β-1a in combination with corticosteroids could increase mortality in ARDS.

Since the first clinical description of ARDS, there has been a great interest in the role of corticosteroids for attenuating the underlying inflammatory state of ARDS because of their potent anti-inflammatory and antifibrotic effects on multiple signaling pathways (Rhen and Cidlowski, 2005). Several doses and types of corticosteroids have been evaluated in the context of ARDS with inconclusive results (Annane et al., 2017). Nevertheless, it is plausible that corticosteroids may benefit ARDS in the early stages of the disease process, a situation that was not evaluated in most RCTs. In addition, dexamethasone was never evaluated in a randomized controlled fashion in ARDS patients, despite its potent anti-inflammatory and weak mineralocorticoid effects. Dexamethasone is 20–30 times more potent than the naturally occurring hormone cortisol and 4–5 times more potent than prednisolone (Rhen and Cidlowski, 2005). In addition, dexamethasone has long-lasting pharmacological effects, allowing for a regime of one dose per day (Meijvis et al., 2011). The beneficial effects of dexamethasone in ARDS were unknown until recently. The DEXA-ARDS (*Dexamethasone in ARDS*) trial, was a multicenter, RCT performed in 277 patients with established moderate-to-severe ARDS (defined by a PaO_2_/FiO_2_ ≤ 200 assessed with a PEEP ≥ 10 cmH_2_O and FiO_2_ ≥ 0.5 at 24 h after diagnosis of ARDS) (Villar et al., 2020). Patients were randomized to receive treatment with dexamethasone (20 mg once daily from days 1 to 5, which was reduced to 10 mg from days 6 to 10) or continued routine intensive care (control group). The primary outcome was VFDs, and the secondary outcome was mortality at 60 days after randomization. Treatment with dexamethasone markedly increased the number of VFDs and decreased the risk of 60-day mortality by an absolute 15%. These findings paved the road of using steroids to improve survival among critically ill patients with coronavirus disease 19 (COVID-19) (WHO Rapid Evidence Appraisal for Covid-19 Therapies (React) Working Group et al., 2020).

## Future Trial Design: Personalized Mechanical Ventilation

The history of interventional clinical trials for ARDS is fraught with many failures and only a few successes in the last decade. Since there is no typical ARDS patient (Villar, 2011), the risk of developing ARDS depends on the underlying disease process but also augments with the number of predisposing factors. Ideally, therapies in ARDS should be personalized to the specific predisposing clinical condition or mechanism of organ injury at any given point in time, rather than being provided uniformly to all patients. The most critical factor in managing ARDS patients is the initiation of lung-protective ventilation instantaneously after endotracheal intubation. However, the development of therapeutic strategies for ARDS is complicated because ARDS is not a disease but a very heterogeneous syndrome (Juschten et al., 2021). The optimal ventilation strategy for ARDS still remains to be refined. For example, the physiological meaning of the end-expiratory transpulmonary pressure could be very useful in managing patients with increased chest wall stiffness or to set optimal PEEP during ventilation and weaning in morbidly obese patients (Kacmarek et al., 2021).

Current lung-protective MV strategies have not significantly decreased ARDS-associated all-cause mortality since the ARDSnet trial (Acute Respiratory Distress Syndrome Network, Brower et al., 2000), possibly because those strategies are reserved to ventilating heterogeneous atelectatic and stable lungs in severe ARDS (Nieman et al., 2020). Furthermore, in most RCTs, an unselected, mixed population of ARDS have been studied, missing the opportunity to test whether the experimental MV approach or adjunctive/pharmacological therapy is beneficial in patients having a single etiology or after assessing their “true” degree of hypoxemia under standardized ventilatory settings prior to randomization (Villar et al., 2007). It is highly plausible that in a substantial proportion of patients in recent RCTs in ARDS, the severity of lung injury was modest. Current definition for ARDS (Ranieri et al., 2012) did not require any specific FiO_2_, and only a minimum PEEP level of 5 mH_2_O to calculate the PaO_2_/FiO_2_ for stratifying patients as mild, moderate or severe ARDS. Any change in PEEP or FiO_2_ can result in a modification of lung imaging and PaO_2_/FiO_2_ ratio. Therefore, depending on the applied MV approach, patients may or may not meet criteria for the diagnosis of ARDS. For example, a septic patient with a PaO_2_ of 69 mmHg on a FiO_2_ of 0.35 and 5 cmH_2_O of PEEP satisfies the criteria for moderate ARDS, but there is no need to enroll this type of patients in a trial testing the effects of high PEEP levels and RMs. In addition, systemic inflammation seen in ARDS patients is not specific for ARDS, especially in patients with sepsis, and MV -although it could be considered a curative therapeutic approach in critically ill patients- it is not the cure for sepsis!

Medicine cannot always be provided by a protocol (Florio et al., 2020). Hopefully, it is plausible that in the near future, we will have mechanisms to identify classes or subclasses of ARDS patients that might respond to targeted therapy, including MV, adjunctive therapies, or pharmacological approaches (Sevransky et al., 2021). Research breakthroughs are not enough. To date, attempts to personalize or stratify the care of ARDS patients enrolled in clinical trials using physiologic measures have not been successful, except in the case of the DEXA-ARDS trial where the authors used an enrichment strategy under standardized ventilatory settings to identify patients for enrollment (Villar et al., 2020). If patients enrolled in a trial have a low risk of the condition to prevent, the trial –irrespective of sample size- will not validate the value of the intervention under study (Villar et al., 2005). MV for patients with ARDS would be personalized based on etiology, lung physiology and morphology, and clinical and biological classes or subclasses because explicit numerical values might not apply to individual patients (Villar et al., 2019; Pelosi et al., 2021). Optimization of patient selection is central to the likelihood of success in future trial design for ARDS (Villar et al., 2020; Pelosi et al., 2021). Both prognostic and predictive enrichment strategies can improve the signal-to-noise ratio, allowing smaller sample sizes and increased effects sizes (Villar et al., 2019; Ware et al., 2020).

## Author Contributions

JV drafted the first version of the manuscript. JV, CF, GT, and FS-S contributed to the initial concept and design of this review. JV, CF, GT, LB, PR-S, and FS-S critically revised the manuscript. JV, CF, and PR-S obtained funding for the study. All authors read and approved the final manuscript.

## Conflict of Interest

This manuscript is an investigator-initiated, academic, non-industry sponsored review article, commissioned by Gary F. Nieman, a designated editor for this specific issue on “Protecting the Acutely Injured Lung: Physiologic, Mechanical, Inflammatory, and Translational Perspectives” in Frontiers in Physiology. JV have received a research grant from Getinge for conducting a clinical trial on mechanical ventilation. LB received grants from “Fast Grants for COVID-19 research” at Mercatus Center of George Mason University and from iNO Therapeutics LLC. The remaining authors declare that the research was conducted in the absence of any commercial or financial relationships that could be construed as a potential conflict of interest.

## Publisher’s Note

All claims expressed in this article are solely those of the authors and do not necessarily represent those of their affiliated organizations, or those of the publisher, the editors and the reviewers. Any product that may be evaluated in this article, or claim that may be made by its manufacturer, is not guaranteed or endorsed by the publisher.
